# Digital PCR-based genetic profiling from vitreous fluid as liquid biopsy for primary uveal melanoma: a proof-of-concept study

**DOI:** 10.1186/s13046-025-03374-y

**Published:** 2025-04-17

**Authors:** R. J. Nell, M. Versluis, N. V. Menger, M. C. Gelmi, T. H.K. Vu, R. M. Verdijk, G. P.M. Luyten, M. J. Jager, P. A. van der Velden

**Affiliations:** 1 Department of Ophthalmology, Leiden University Medical Center, Leiden, the Netherlands; 2 Department of Pathology, Leiden University Medical Center, Leiden, the Netherlands; 3 Department of Pathology, Section Ophthalmic Pathology, Erasmus MC University Medical Center, Rotterdam, the Netherlands

## Abstract

**Background:**

Uveal melanoma is an aggressive ocular malignancy. Early molecular characterisation of primary tumours is crucial to identify those at risk of metastatic dissemination. Although tumour biopsies are being taken, liquid biopsies of ocular fluids may form a less invasive but relatively unexplored alternative. In this study, we aim to evaluate the DNA content of vitreous fluid from eyes with a uveal melanoma to obtain molecular tumour information.

**Methods:**

DNA was isolated from 65 vitreous fluid samples from enucleated eyes with a uveal melanoma and studied using digital PCR. Primary and additional driver mutations (in *GNAQ*, *GNA11*, *PLCB4*, *CYSLTR2*, *BAP1*, *SF3B1* and *EIF1AX*) were investigated using accustomed targeted and drop-off assays. The copy numbers of chromosome 3p and 8q were measured using multiplex and single-nucleotide polymorphism-based assays. Our findings were compared to the molecular profile of matched primary tumours and to the clinicopathological tumour characteristics.

**Results:**

Almost all (63/65) vitreous fluids had measurable levels of DNA, but melanoma-cell derived DNA (containing the primary driver mutation) was detected in 45/65 samples (median proportion 15.5%, range 0.03-94.4%) and was associated with a larger tumour prominence, but not with any of the molecular tumour subtypes. Among the vitreous fluids with melanoma-cell derived DNA, not all samples harboured (analysable) other mutations or had sufficient statistical power to measure copy numbers. Still, additional mutations in *BAP1*, *SF3B1* and *EIF1AX* were detected in 15/17 samples and chromosome 3p and 8q copy numbers matched the primary tumour in 19/21 and 18/20 samples, respectively. Collectively, a clinically-relevant molecular classification of the primary tumour could be inferred from 29/65 vitreous fluids.

**Conclusions:**

This proof-of-concept study shows that substantial amounts of DNA could be detected in vitreous fluids from uveal melanoma patients, including melanoma-cell derived DNA in 69% of the samples. Prognostically-relevant genetic alterations of the primary tumour could be identified in 45% of the patients. A follow-up study is needed to evaluate our approach in a prospective clinical context. Additionally, our work highlights improved possibilities to sensitively analyse scarce and heterogeneous tumour biopsies, with potential application in other malignancies.

**Supplementary Information:**

The online version contains supplementary material available at 10.1186/s13046-025-03374-y.

## Background

**Fig. 1:**
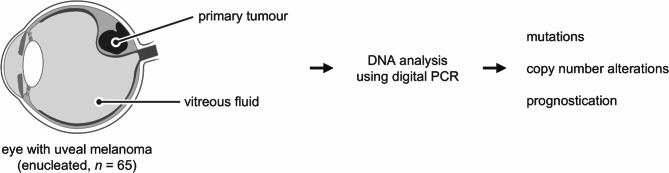
Workflow of current study: DNA from primary tumours [20] and vitreous fluid samples in 65 enucleated eyes is analysed by digital PCR.

Uveal melanoma is a rare, aggressive intraocular tumour originating from malignantly-transformed melanocytes in the iris, ciliary body or choroid (Fig. 1). The majority of uveal melanomas are located in the relatively inconspicuous and inaccessible posterior segment of the eye [1]. Consequently, primary tumours can be large upon first detection and (micro)metastases may occur before first treatment [2, 3]. For that reason, early and adequate identification and characterisation of primary uveal melanomas is crucial to prevent tumour progression and ameliorate patient outcome.

Over the past decades, various recurrent genetic alterations have been identified in primary uveal melanomas. A Gα_q_ signalling mutation at an established hotspot in *GNAQ*, *GNA11*, *CYSLTR2* or *PLCB4* is considered the primary driver event and is present in nearly all tumours. Prognostically-relevant molecular tumour subtypes can be distinguished based on the presence of additional drivers: a so-called ‘BSE mutation’ in *BAP1*, *SF3B1* or *EIF1AX* and copy number alterations (CNAs) affecting chromosome 3p or 8q. Uveal melanomas with *EIF1AX* mutations are generally copy number stable and have the best prognosis with the lowest risk of early metastatic dissemination [4, 5]. *SF3B1*-mutant tumours often present with additional copies of chromosome 8q and are associated with an intermediate prognosis with relatively late metastases [4, 6–8]. Uveal melanomas with a loss of chromosome 3p and a *BAP1* mutation, typically also showing chromosome 8q copy number increases, have the poorest prognosis [9, 10].

Primary uveal melanoma is usually treated by local, eye-preserving procedures (brachytherapy, proton beam therapy) or a complete surgical removal of the eye (enucleation) [1]. In case of the latter, tumour tissue is readily available for molecular analysis, and such analysis is usually offered to all enucleated patients. For non-enucleated patients, however, an additional tumour biopsy is required to obtain a molecular profile of the melanoma. While intra-ocular fine-needle aspirate tumour biopsies are being taken routinely in a number of clinics, the procedure is relatively difficult (requiring vitreoretinal surgical expertise), invasive and remains accompanied by various risks, such as retinal detachment, intraocular haemorrhage, and potentially intraocular infection and tumour seeding [11, 12]. A less invasive (outpatient) biopsy procedure would allow more patients to have a personalised molecular diagnosis and prognosis. Moreover, those with an increased risk of metastatic dissemination may be offered a more intense follow-up screening program, and could possibly benefit from earlier detection of metastasis. Additionally, knowledge about the molecular profile of the primary tumour has been positively associated with various aspects of psychosocial well-being [13] and might be of (future) clinical relevance with regard to novel (neo-)adjuvant or targeted therapies [5, 14, 15].

Previously, we introduced digital PCR assays to analyse the key genetic alterations in uveal melanoma to quantify the intratumour heterogeneity in bulk tumour specimens [16–20]. Digital PCR can also be used for the sensitive detection of genetic alterations in scarce DNA samples, such as liquid biopsies (i.e. samples of body fluids such as blood or serum, urine or aspirates) [21]. In this study (Fig. 1), we isolate DNA from 65 vitreous fluid samples (referred to as vfDNA) obtained from enucleated eyes with a uveal melanoma. These samples are analysed for mutations in *GNAQ*, *GNA11*, *PLCB4*, *CYSLTR2*, *EIF1AX*, *SF3B1* and *BAP1*, and copy number alterations affecting chromosomes 3p and 8q. Next, our findings are compared to the molecular profile of matched primary tumours and to the clinicopathological tumour characteristics. Collectively, this study forms the proof-of-concept for a comprehensive characterisation of the DNA content of the vitreous as a potential liquid biopsy for uveal melanoma patients to identify those at high risk of developing metastases.

## Materials and methods

### Vitreous fluid sample collection and DNA isolation

Vitreous fluid samples were collected from 65 enucleated eyes with a uveal melanoma as part of the biobank of the Department of Ophthalmology, Leiden University Medical Center (LUMC). Samples had been isolated by syringe suction directly after opening the enucleated eye, and stored at −80 °C until DNA isolation (to prevent DNA degradation no repeated freeze-thaw cycles were carried out). This study was approved by the LUMC Biobank Committee and Medisch Ethische Toetsingscommissie under numbers B14.003/SH/sh and B20.026/KB/kb.

Total DNA was isolated from 100 to 200 uL vitreous fluid using the Quick-cfDNA Serum & Plasma Kit (Zymo Research, Irvine, USA) and eluted in 35 uL DNA elution buffer (Zymo), following the manufacturer’s instructions. From all samples, the matched primary uveal melanoma was analysed in our recent study using digital PCR and targeted DNA and RNA sequencing [20]. Clinical and molecular information of all samples is summarised in Supplementary Table 1.

### Digital PCR experiments

All digital PCR experiments were carried out using the QX200 Droplet Digital PCR System (Bio-Rad Laboratories, Hercules, USA) using the assays and following the protocols described earlier [16–20, 22]. Typically, 3.5 uL isolated vfDNA was analysed in a single 22 uL experiment, using 11 uL ddPCR Supermix for Probes (No dUTP, Bio-Rad) and primers and FAM- or HEX-labelled probes in final concentrations of 900 and 250 nM for duplex experiments, or optimised concentrations for multiplex experiments. Assay details are described in our recent study [20].

All PCR mixtures were partitioned into 20,000 droplets using the AutoDG System (Bio-Rad). Next, a PCR protocol was performed in a T100 Thermal Cycler (Bio-Rad): 10 min at 95 °C; 30 s at 94 °C and 1 min at 55 °C, 58 °C or 60 °C for 40 cycles; 10 min at 98 °C; cooling at 12 °C for up to 48 h, until droplet reading. Ramp rate was set to 2 °C/second for all steps.

Reading of the droplets was performed using the QX200 Droplet Reader (Bio-Rad), which has in-built sensors to ensure an equal volume of the droplets. Following to the manufacturer’s recommendations, 10,000 was set as the minimum number of accepted droplets to pass the quality control. Thresholding was performed manually via two-dimensional plots (FAM versus HEX) in comparison to included positive and negative controls. For all assays, at least 3 positive droplets were required to confirm the detectability of the target tested.

The raw results of the digital PCR experiments were acquired using *QuantaSoft* (version 1.7.4, Bio-Rad) and imported and analysed in the online digital PCR management and analysis application *Roodcom WebAnalysis* (version 2.0, available via https://webanalysis.roodcom.nl).

First of all, the Gα_q_ signalling mutations in *GNAQ*, *GNA11*, *CYSLTR2* and *PLCB4* were analysed in duplex experiments and the measured concentrations (listed between square brackets) of the mutant and wild-type alleles were used to calculate the total vfDNA concentrations and melanoma-cell derived vfDNA fractions:

$$\text{total}\,\text{vfDNA}\,\text{concentration} = [\text{mutation}] + [\text{wildtype]}$$

$$\text{melanoma-cell}\,\text{derived}\,\text{vfDNA}\,\text{fraction} = 2 \cdot \frac{[\text{mutation}]}{[\text{mutation}] + [\text{wildtype}]}$$

Additional BSE mutations in *BAP1*, *SF3B1* and *EIF1AX* were interpreted in a qualitative manner (i.e. ‘detected’ or ‘not detected’). For all Gα_q_ and BSE mutational analyses, initially negative samples were tested in up to three experiments and merged afterwards to increase the likelihood of finding target alleles with a low abundance (summarised in Supplementary Table 1).

CNAs were measured via two different approaches, as validated previously [19]. Following the classic approach, copy number values were calculated based on the measured concentrations of a DNA target on chromosome 3p (*PPARG*) and 8q (*PTK2*), and a reference gene on chromosome 5p (*TERT*) or 14q (*TTC5*) assumed to be stable:

$$\text{copy}\,\text{number}\,\text{value} = 2 \cdot \frac{[\text{target}]}{[\text{reference}]}$$

Following the SNP-based approach, copy number values were measured using the concentrations of haploid genomic loci var_1_ and var_2_, representing the two variants of a heterozygous germline single-nucleotide polymorphism (SNP):

$$\text{copy}\,\text{number}\,\text{value} = 1 + \frac{[\text{var}_1]}{[\text{var}_2]}$$

In this formula, the SNP variant in the denominator (var_2_) is the one that was assumed to be unaltered and copy number stable (i.e. haploid). Analysed SNPs in this study included rs6617, rs6976, rs9586, rs1989839, rs1062633 and rs2236947 on chromosome 3p, and rs7018178 and rs7843014 on chromosome 8q. For all vitreous fluid patients, genotyping and validation of the SNP assays was performed in the matched primary uveal melanoma in our recent study [20].

### In silico evaluation of digital PCR sensitivity and power prediction

To evaluate the statistical power of detecting CNAs in the vfDNA+/UM + samples, we performed in silico digital PCR simulations using our R library *digitalPCRsimulations* (available via https://github.com/rjnell/digitalPCRsimulations), as described recently [19]. In short, for each individual sample, we determined the sensitivity (% of simulated experiments) of detecting a chromosomal loss (copy number = 1) or gain (copy number = 3), assuming that this alteration is clonally present in the entire vfDNA melanoma-cell population. An alteration was considered ‘detected’ when the obtained copy number was lower or higher than 2 and the constructed 99%-confidence interval did not contain 2. The input vfDNA concentration and melanoma-cell fraction were based on the initial Gα_q_ mutation and wild-type measurements. The total number of accepted droplets was set to 15,000 (for a single experiment) and each condition was simulated up to 1,000 times. An experimental setup (classic or SNP-based approach) was considered ‘underpowered’ in samples with a sensitivity < 80% when analysing a maximum of 45,000 droplets (representing a merge of three experiments).

### Statistical analysis and code availability

Statistical evaluations in this study were conducted using Spearman’s rank correlation coefficient, Fisher’s exact test and the Mann-Whitney *U* test due to the relatively small sample sizes and non-normal distribution of the data. The log-rank test was used to test associations with melanoma-related survival. All analyses were performed using R (version 4.0.3) in RStudio (version 1.4.1103) with R library digitalPCRsimulations (version 1.1.1, available via https://github.com/rjnell/digitalPCRsimulations). All custom scripts are available with annotation via https://github.com/rjnell/um-vitreous.

## Results

### DNA composition of vitreous fluid samples assessed by mutant and wild-type Gα_q_

**Fig. 2:**
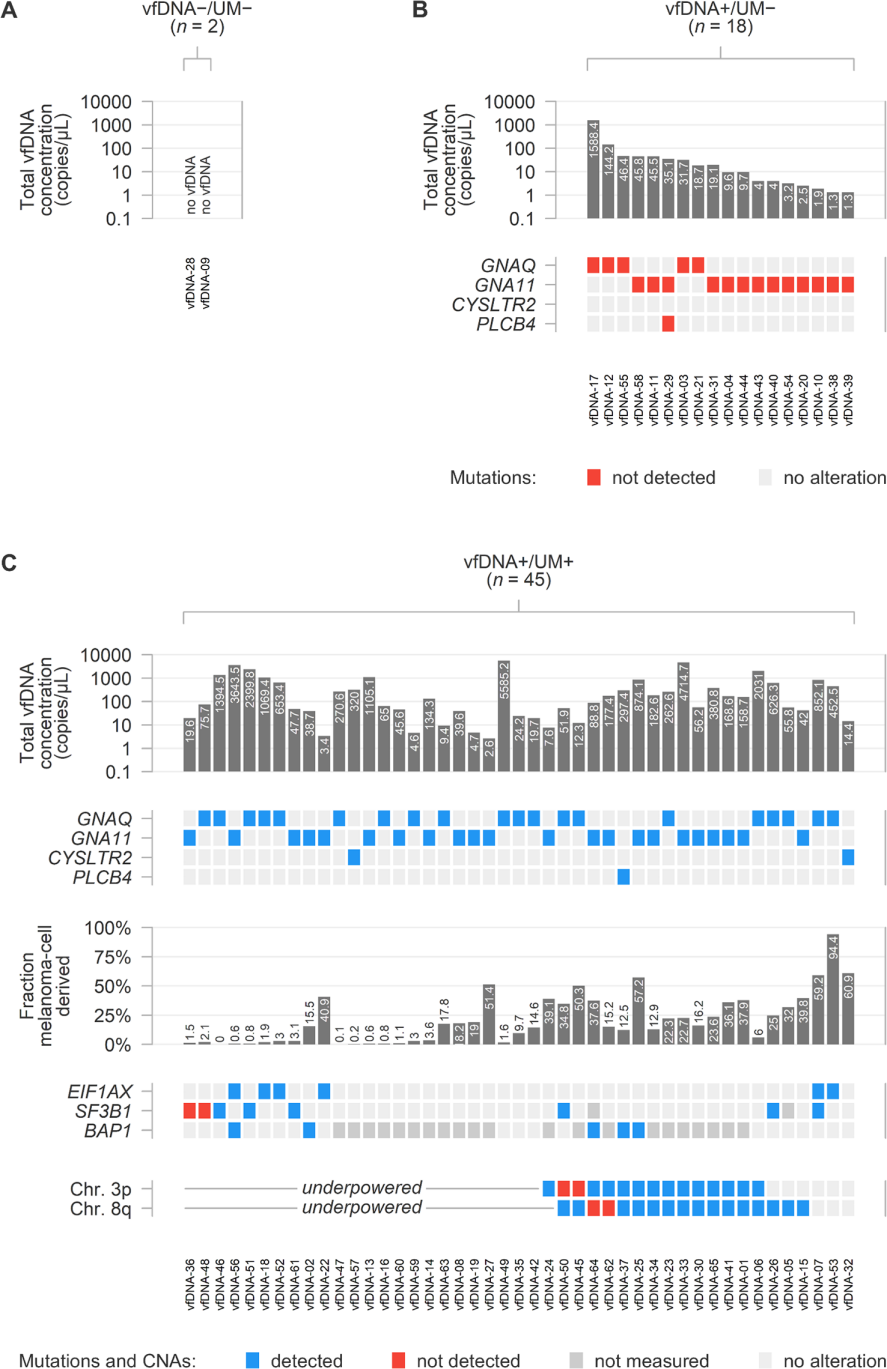
DNA composition and detectability of molecular alterations in 65 vitreous fluid samples. Mutations and CNAs are compared to those identified in the matched primary tumour [20], and their detection in the vfDNA is visualised. (A) 2/65 (3%) samples had no measurable amounts of vfDNA and were labelled vfDNA−/UM−. (B) 18/65 (28%) vitreous fluid samples had measurable levels of wild-type vfDNA but lacked DNA that was derived from mutant melanoma cells. These samples were labelled vfDNA+/UM−. (C) 45/65 (69%) samples contained both wild-type and mutant DNA (derived from melanoma cells) and were labelled as vfDNA+/UM+. In these samples, *EIF1AX* mutations could be identified in 6/6 vitreous fluid samples, *SF3B1* mutations in 6/8 samples and *BAP1* mutations in 5/5 samples 6/6. ‘Not measured’ refers to a selection of mutations that were known to be present in the primary tumour, but could not be analysed in the vfDNA sample due to lack of a dedicated digital PCR assay (*n* = 0 for *EIF1AX*, *n* = 2 for *SF3B1* and *n* = 21 for *BAP1*). ‘Underpowered’ refers to the condition in which the amount of total and melanoma-cell derived vfDNA was predicted to be insufficient to detect chromosomal CNAs (i.e. predicted sensitivity < 80%, *n* = 24 for chromosome 3p and *n* = 25 for chromosome 8q). In the vfDNA+/UM+ samples with sufficient statistical power, the presence of chromosome 3p and 8q alterations matched those in the primary tumour in 19/21 (90%) and 18/20 (90%) samples.

**Fig. 3:**
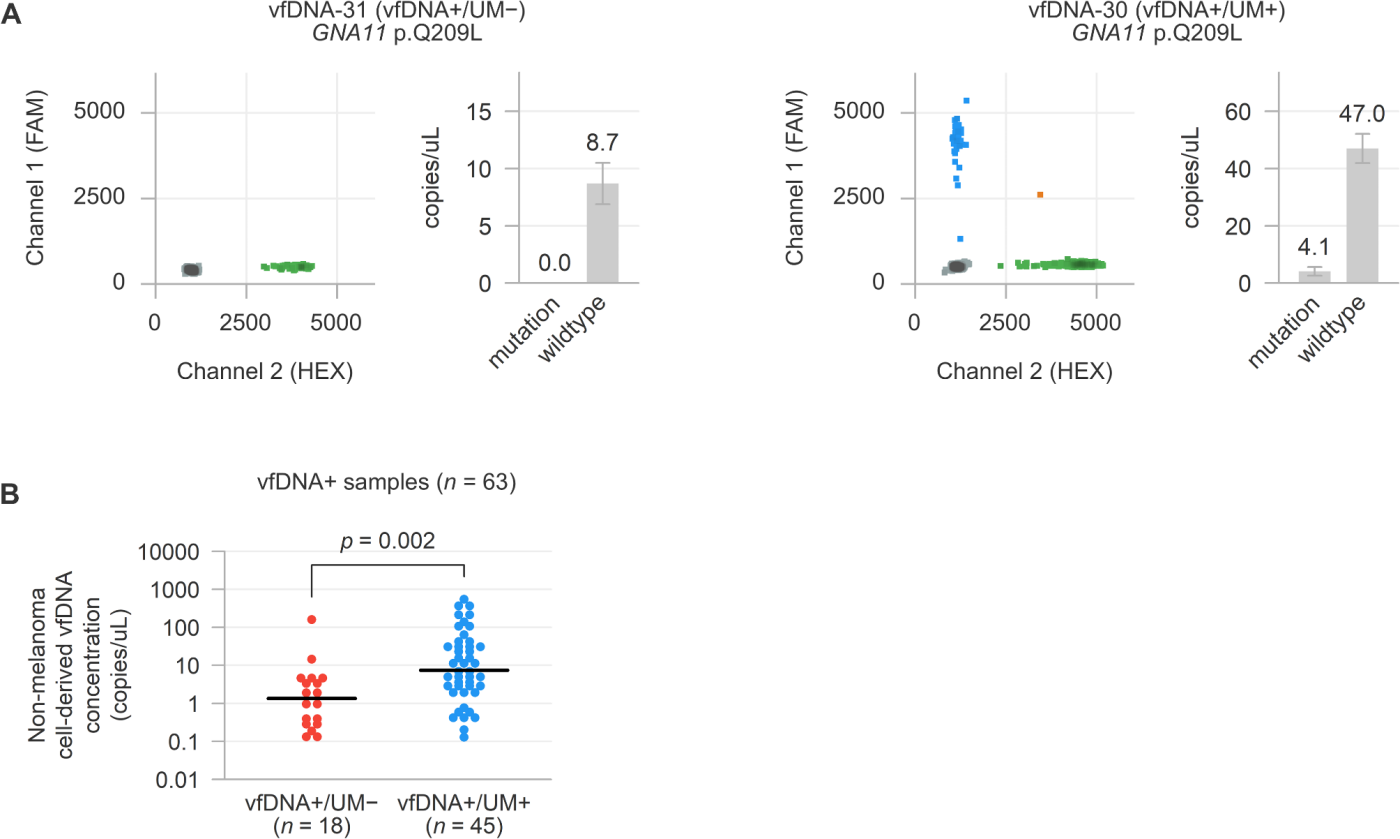
(A) Analysis of Gαq signalling mutations using targeted digital PCR, here exemplified for the measurement of *GNA11* p.Q209L mutant and wild-type DNA alleles with two representative two-dimensional plots for a vfDNA+/UM− and vfDNA+/UM+ sample. The colour of the cluster indicates the content of the digital PCR droplets (grey: no target DNA, blue: mutant DNA, green: wild-type DNA, orange: wild-type and mutant DNA). (B) Comparison of non-melanoma cell-derived DNA concentrations in vfDNA+/UM− and vfDNA+/UM+ samples.

As the Gα_q_ signalling mutations form the most clonal and most common genetic alteration in the primary tumour, they represent a relatively generic marker of the malignant cells and can be used to quantify the abundance of melanoma-cell derived DNA [20]. Based on the exact Gα_q_ mutation that was identified earlier in the matched primary tumour [20], we investigated all vfDNA samples using targeted digital PCR for the absolute and relative abundance of mutant and wild-type DNA (Figs. 2 and 3A). To account for potential subsampling in specimens with a low DNA abundance, initially negative samples were reanalysed in up to three experiments and led to identification of additional positive cases (Supplementary Fig. 1A and B). A variety of vfDNA samples from wild-type tumours were tested as negative controls and turned out to be consistently negative (Supplementary Fig. 1C).

We did not find mutant or wild-type alleles in 2/65 (3%) samples, indicating that these samples did not contain measurable amounts of vfDNA (Fig. 2A). Wild-type Gα_q_ alleles only were detected in 18/65 (28%) vitreous fluid samples; these samples were labelled as vfDNA+/UM−, as they had measurable levels of vfDNA but lacked DNA that was derived from mutant melanoma cells (Figs. 2B and 3A). Still, some of the samples contained large amounts of DNA (median 14.2, range 1.1–1588.4 genomic copies per uL vitreous fluid). In one vfDNA+/UM− patient, the primary uveal melanoma was known to carry two clonally-abundant Gα_q_ signalling mutations (*GNA11* p.R183H and *PLCB4* p.D630N in vfDNA-29), but the vfDNA only contained wild-type *GNA11* and *PLCB4* in comparable concentrations (Supplementary Fig. 1D).

Mutant Gα_q_ alleles were detected in 45/65 (69%) samples, which were labelled as vfDNA+/UM+ (Figs. 2C and 3A). In these samples, the sum of mutant and wild-type allele concentrations was taken as total vfDNA concentration (median 134.3, range 2.6–5585.3 genomic copies per uL). The mutant allele fractions were used to estimate the abundance of melanoma-cell derived vfDNA, revealing proportions between 0.03% and 94.2% (median 15.5%). vfDNA+/UM+ samples also contained significantly more non-melanoma cell derived vfDNA (i.e. DNA not derived from uveal melanoma cells, Mann-Whitney U *p* = 0.002) than vfDNA+/UM− samples (Fig. 3B).

### Analysis of additional BSE mutations in *BAP1*, *SF3B1* and *EIF1AX*

**Fig. 4:**
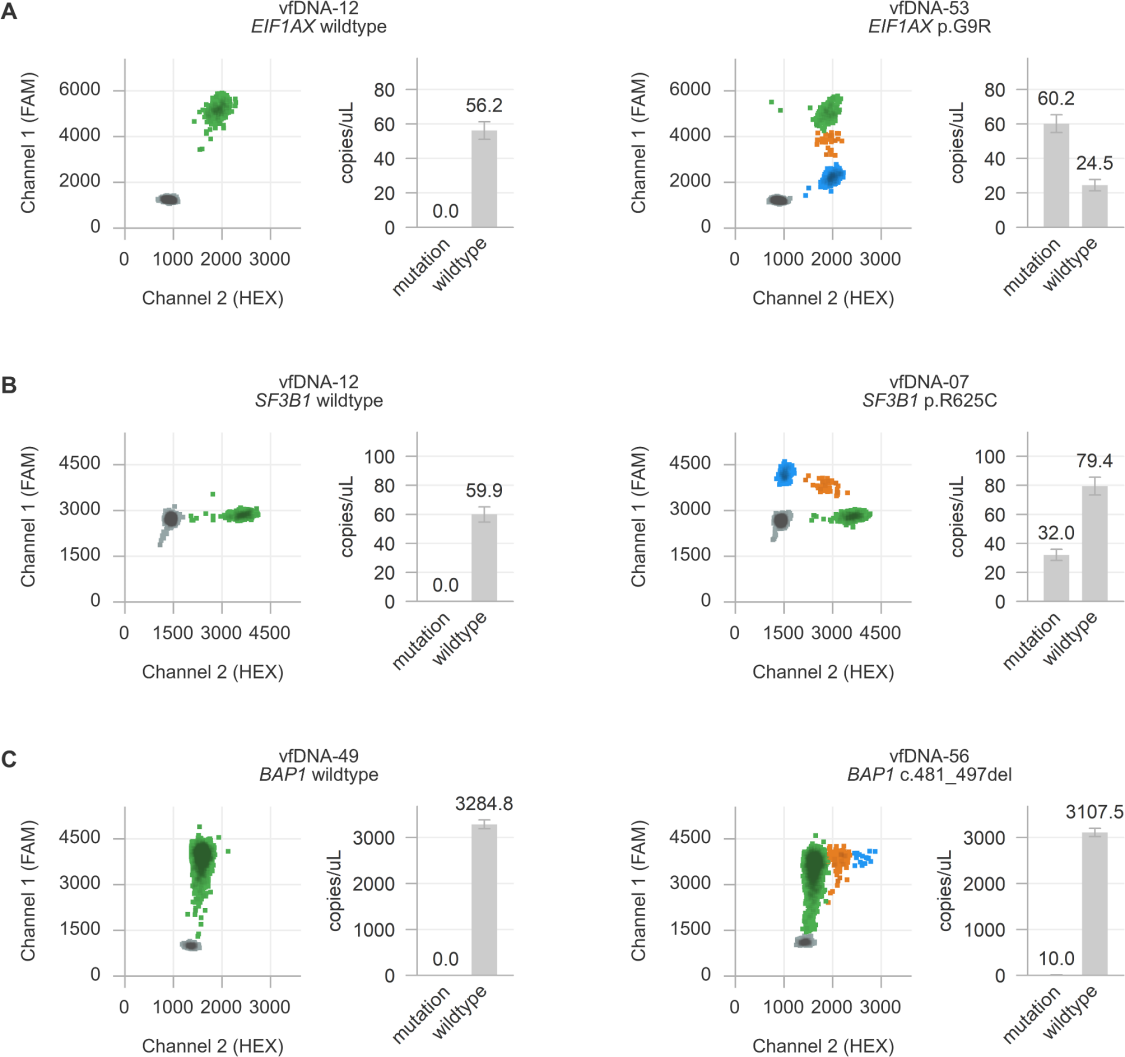
Analysis of BSE mutations in *EIF1AX* (A), *SF3B1* (B) and *BAP1* (C) using drop-off and targeted digital PCR assays. Here exemplified with representative two-dimensional plots for a wild-type and mutant vfDNA sample with roughly comparable vfDNA concentrations. The colour of the cluster indicates the content of the digital PCR droplets (grey: no target DNA, blue: mutant DNA, green: wild-type DNA, orange: wild-type and mutant DNA).

In contrast to the primary Gα_q_ mutations, only a subset of the additional BSE mutations can be found at a hotspot position. In our cohort of primary uveal melanomas from vfDNA+/UM+ patients [20], *BAP1* alterations occurred throughout the entire gene, most of the *SF3B1* mutations affected position p.R625, and *EIF1AX* mutations were found at various positions within the first two exons of the gene (Supplementary Table 1). To be able to analyse these diverse BSE mutations, we developed a variety of targeted and generic drop-off digital PCR assays [20]. Hereby, a small number of unique *BAP1* alterations, all hotspot mutations in *SF3B1* (p.R625C/H), and all mutations in *EIF1AX* (exon 1 and exon 2) could be analysed (Fig. 4 and Supplementary Table 1). We now applied these assays in the corresponding cohort of vfDNA+/UM+ samples (*n* = 17, Fig. 2C). Similar to the Gα_q_ mutations, a (merged) analysis of up to three experiments was carried out to account for subsampling, and a selection of vfDNA+/UM− samples with matched vfDNA concentrations were successfully measured as negative controls (Fig. 4 and Supplementary Fig. 2A and B).

Based on the known mutational status of the respective primary tumours, *EIF1AX* mutations were successfully identified in 6/6 vitreous fluid samples, *SF3B1* mutations in 6/8 samples and *BAP1* mutations in 5/5 samples (Fig. 2). Two samples (vfDNA-07 and vfDNA-56) carried two BSE mutations, which were both detected in the vfDNA in comparable concentrations. Of note, the two vitreous fluid samples with undetected *SF3B1* mutation (vfDNA-36 and vfDNA-48) had two of the lowest concentrations of Gα_q_ mutant alleles within the vfDNA+/UM+ cohort (Fig. 2C and Supplementary Table 1). In total, 15/17 (88%) of the analysed vitreous fluid samples had a measurable BSE mutation matching with the corresponding primary tumour.

### Advanced chromosome 3p and 8q copy number analysis

**Fig. 5:**
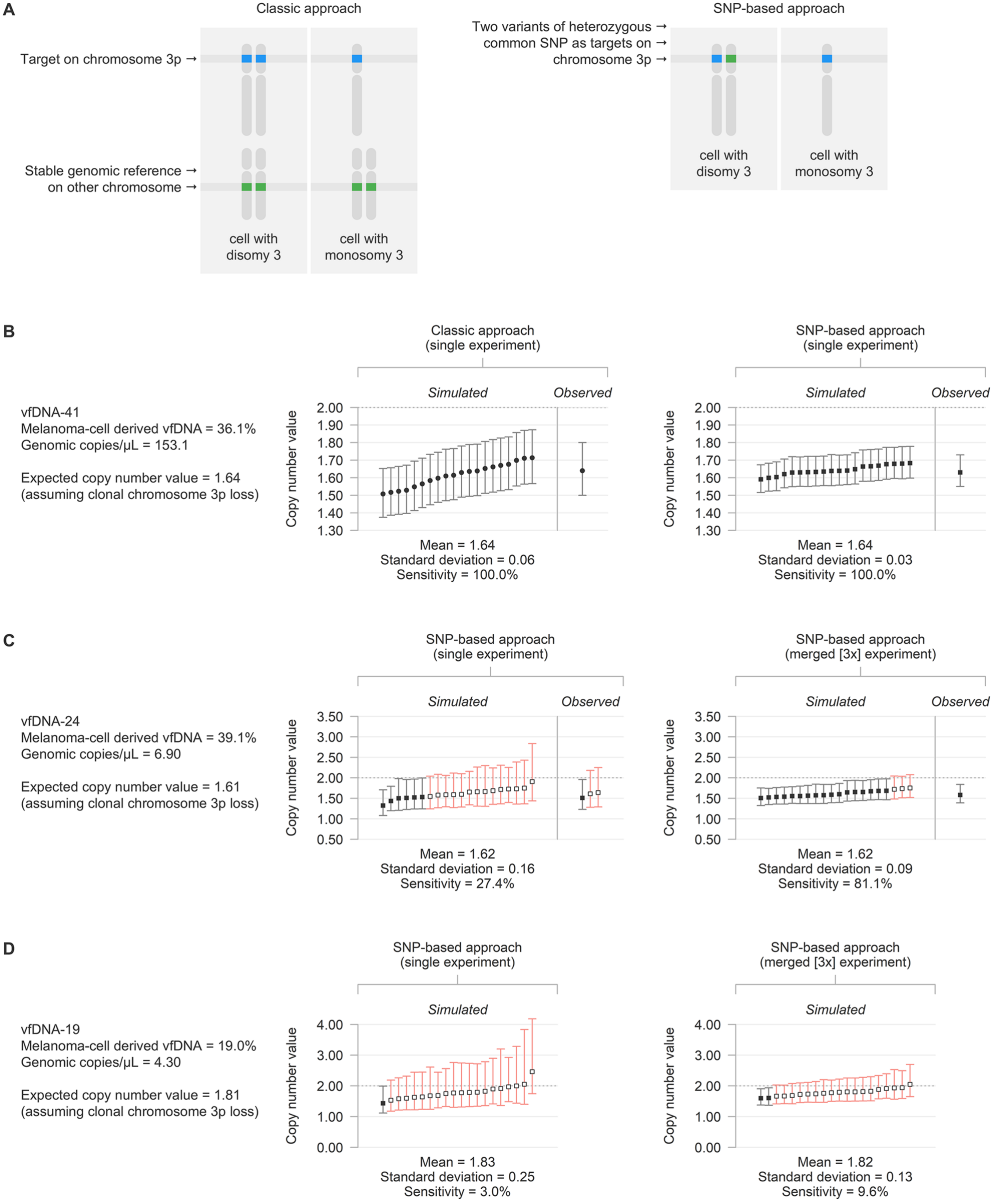
(**A**) Approaches to analyse copy number alterations, here exemplified for measuring chromosome 3p loss. (**B**) Simulated and observed copy number value measurements for chromosome 3p in vfDNA-41, with associated 99%-confidence intervals. Simulations were based on the initial Gα_q_ mutant and wild-type measurements and the assumption of a clonal loss in the vfDNA melanoma-cell population, in single experiments using both the classic and SNP-based approach. Significance of the measurements is indicated by the symbol and colour of the individual confidence interval (empty symbol and red colour: not significantly different from 2 [*p* > 0.01], filled symbol and grey colour: significantly different from 2 [*p* < 0.01]). (**C**) Overview of simulated and observed copy number value measurements for chromosome 3p in vfDNA-24, in both single and (3x) merged experiments using the SNP-based approach. (**D**) Overview of simulated copy number value measurements for chromosome 3p in vfDNA-19, in both single and (3x) merged experiments using the SNP-based approach.

CNAs are usually studied by comparing the absolute abundance of a target of interest to a stable genomic reference (classic approach, Fig. 5A) [19]. As an alternative, CNAs may also be measured by evaluating the allelic (im)balance between the two variants of a heterozygous germline SNP on the chromosome of interest (SNP-based approach, Fig. 5A). While both SNP variants are equally abundant under healthy conditions, this balance is altered when there is a copy number loss or gain of one of the alleles. Conveniently, a stable genomic reference is not required when using this approach and more precise measurements can be obtained. We recently validated this methodology for detecting chromosome 3p and 8q alterations in uveal melanomas, including the primary tumours from the cohort analysed in this study [19, 20]. In line with our earlier analyses in tumour DNA, the SNP-based approach was generally more precise than the classic approaches in the vfDNA samples as well (exemplified in Fig. 5B), and was chosen as preferred methodology whenever available (Supplementary Table 1).

The precision of digital PCR measurements also depends on the amount of DNA input (limited by the low quantity of our samples) and the number of partitions (i.e. droplets) [19]. A typical digital PCR experiment yields between 10,000 and 20,000 accepted droplets, but this can be increased by repeating the experiment and merging the results into one combined analysis. The limiting factor, however, remains that those experimental replicates also require more sample DNA. To determine the statistical power of detecting CNAs in our application of analysing samples with diverse total and melanoma-cell derived vfDNA concentrations, we performed in silico simulations [19] based on the initial Gα_q_ mutant and wild-type measurements of all vfDNA+/UM+ samples. For each individual sample, we evaluated the sensitivity (i.e. the estimated proportion significant experiments) of detecting a chromosomal loss (copy number = 1) or gain (copy number = 3) assuming that this alteration is clonally present in the vfDNA melanoma-cell population. As representative examples, the in silico analyses and in vitro observations for measuring chromosome 3p loss in three vitreous fluid samples are presented in Fig. 5B-D. A summary of the analyses for all cases is available in Supplementary Table 1.

Some vfDNA samples were predicted to contain enough DNA to detect a chromosomal alteration in a single digital PCR experiment (as illustrated by vfDNA-41, Fig. 5B). In other samples, the sensitivity of detecting a CNA was lower, but this could be increased by repeating the experiment up to three times and combining the results (such as in vfDNA-24, Fig. 5C). In the remaining group of samples, the power of detecting a CNA was so low, that even merging three experiments was not sufficient to lead to (potentially) significant results (exemplified by vfDNA-19, Fig. 5D). In general, higher levels of sensitivity were obtained when more droplets could be analysed, when more melanoma-cell derived vfDNA was present and when less healthy cell DNA ‘contaminated’ the sample (Supplementary Table 1).

Based on the in silico results, we limited further copy number analysis to those vitreous fluid samples with an expected sensitivity > 80% of calling a loss (of chromosome 3p, in 21/45 [47%] of the vfDNA+/UM+ samples) or gain (of chromosome 8q, in 20/45 [44%] of the vfDNA+/UM+ samples) in the available amount of vfDNA (**Materials and methods**, Fig. 2C and Supplementary Table 1). Within this subgroup, chromosome 3p losses were detected in 13/15 samples with a known loss in the matched primary tumour. Similarly, chromosome 8q copy number increases were detected in 15/17 samples with a known chromosome 8q CNA in the tumour. It is noteworthy that in all four discordant cases, the CNA (that remained undetected in the vfDNA) was heterogeneously present in the matched primary tumour: two uveal melanomas had a subclonal loss of chromosome 3p and two a subclonal gain of chromosome 8q (Supplementary Fig. 3 and Supplementary Table 1) [20]. We did not detect CNAs in vfDNA+/U+ samples with no chromosome 3p (*n* = 6) or 8q (*n* = 3) alterations in the matched primary tumour, nor in a selection of underpowered vfDNA+/UM+ samples and vfDNA+/UM− samples (which lack copy number altered melanoma-cell derived DNA). Overall, the presence of chromosome 3p and 8q alterations matched those in the primary tumour in 19/21 (90%) and 18/20 (90%) analysable and selected vitreous fluid samples, respectively.

### Clinicopathological context

**Fig. 6:**
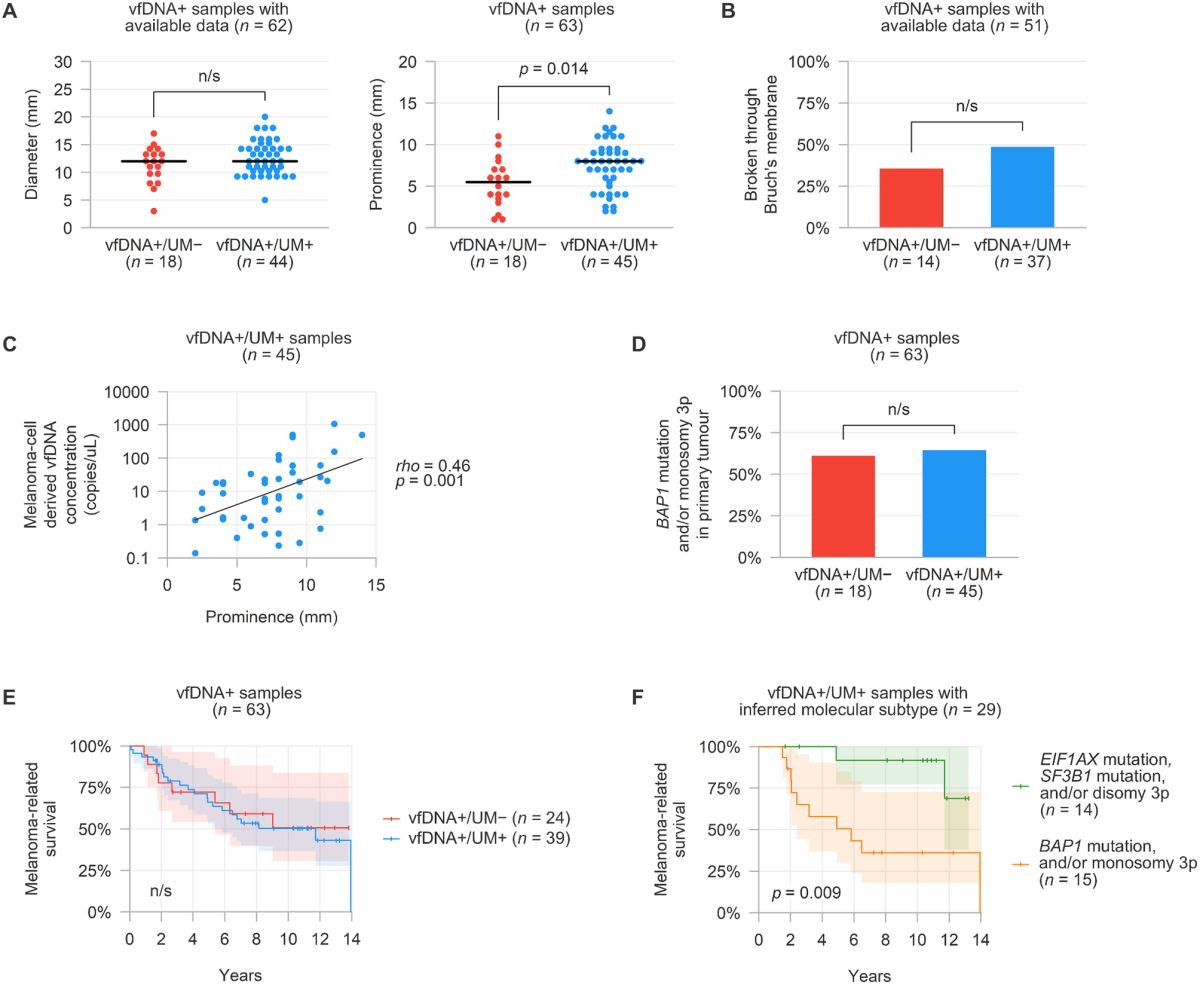
(**A**) Comparison in pathologically-determined largest basal diameter and prominence of matched primary tumours of vfDNA+/UM− or UM+ samples. The black horizontal line denotes the median value for both groups. (**B**) Comparison in frequency of pathologically-determined breaching of Bruch’s membrane in matched primary tumours of vfDNA+/UM− or UM+ samples. (**C**) Correlation between concentrations of mutant vfDNA and pathologically-determined prominence of matched primary tumours in vfDNA+/UM+ samples. (**D**) Comparison in frequency of matched primary tumours with a poor prognostic molecular subtype (*BAP1* mutation and/or monosomy 3p) of vfDNA+/UM− or UM+ samples. (**E**) Melanoma-related survival of patients within the vfDNA+/UM− or UM+ groups. (**F**) Melanoma-related survival of patients based on the inferred molecular subtypes from vfDNA measurements.

Lastly, we investigated whether the vfDNA content correlated to the clinicopathological context of the primary tumour. We hypothesised that the detectability of melanoma-cell derived vfDNA related to anatomical characteristics of the primary tumour (Fig. 6A and B). No significant differences were found with regard to the largest basal diameter and frequency tumours broken through Bruch’s membrane. However, patients with melanoma-cell derived vfDNA had a larger prominence of their primary tumour (median 8.0 versus 5.5 mm, Mann-Whitney U *p* = 0.014) than those without. Within the vfDNA+/UM+ samples, there was a significant positive correlation between the prominence of primary tumour and concentration of melanoma-cell derived vfDNA (Spearman’s rho = 0.46, *p* = 0.001, Fig. 6C).

Based on the genetic analysis of the matched primary tumours [20], the distribution of the various molecular uveal melanoma subtypes within the vfDNA+/UM− and vfDNA+/UM+ patients was found to be very similar (Fig. 6D and Supplementary Table 1). Not surprisingly, the absence or presence of melanoma-cell derived vfDNA itself did not show an association with melanoma-related survival (Fig. 6E). Focussing on the subsequent vfDNA measurements, however, the molecular subtype of the primary tumour could be successfully inferred from 29/65 (45%) of the vfDNA samples. Within this subgroup, the prognostic relevance of the molecular classification was evident (log-rank *p* = 0.009, Fig. 6F): patients with a *BAP1* mutation and/or monosomy 3p had a median melanoma-related survival of 5.8 years, while the estimated median survival was not reached in the group of patients with an *EIF1AX* mutation, *SF3B1* mutation and/or disomy 3p.

## Discussion

In various malignancies, the analysis of liquid biopsies has proven to provide clinically-relevant molecular information of primary or metastatic tumours, while forming a less invasive alternative to direct tumour biopsies [21, 23]. In ocular oncology, the genetic analysis of eye fluids has a prominent role in the differentiation between a malignant intra-ocular lymphoma and a benign uveitis [24], but it remained unexplored in the context of characterising uveal melanoma. For this reason, we investigated the DNA from the vitreous fluid as a potential source of molecular information from the primary tumour, using digital PCR (Fig. 1).

Firstly, we measured the total amount and fraction of melanoma-cell derived vfDNA based on the abundance of mutant and wild-type Gα_q_ alleles (Figs. 2 and 3). This analysis was successful in 97% of the samples, indicating that nearly all vitreous fluids contained measurable levels of DNA. However, in only 69% of all samples part of this genetic material was derived from melanoma cells. This implies that a considerable proportion of the DNA present in the vitreous originated from non-malignant cells. It would be interesting to further characterise the cell type of origin of this DNA, for example by applying genetic immune cell measurements or epigenetic profiling [25–28].

Next, we tested the vfDNA+/UM+ samples (i.e. the vitreous fluids containing melanoma-cell derived DNA) for additional DNA alterations. Based on the known genetic profile of the matched primary tumour, 15/17 (88%) of the analysed vitreous fluid samples had a measurable BSE mutation (Figs. 2 and 4). Importantly, however, this supervised mutational screening is not feasible when no matched tumour material is available. In such situations, the vfDNA should be screened for all possible alterations, which requires multiple experiments and is sample-expensive. A possible solution may be found in the development of multiplex setups in which various assays are combined within one experiment. Our *EIF1AX* drop-off assays furthermore proved that mutation analyses can be made more generic: all different exon 1 and 2 mutations could be measured with only two distinct assays. Similar drop-off approaches may be developed for the Gα_q_ signalling mutations and the majority of *SF3B1* mutations. However, it is unlikely that comparable assays can be developed to generically detect all *BAP1* mutations, as these mutations do no occur at hotspot positions and are practically unique in each mutant tumour.

In addition to the BSE mutations, CNAs were studied in the vfDNA+/UM+ samples. The presence of chromosome 3p and 8q alterations corresponded with those in the primary tumour in 19/21 (90%) and 18/20 (90%) of the analysable cases, respectively (Figs. 2 and 5). Though, we noted that remarkable differences exist in the detectability of melanoma-cell derived mutations versus CNAs: whereas the qualitative identification of few mutant alleles inevitably confirms the presence of this mutation, a CNA can only be detected upon a precise quantification of the chromosomal target in comparison to the healthy copy number invariant situation. This leads to sample-specific detection limits that are higher than those for mutations, especially in samples with large amounts of ‘contaminating’ unaltered DNA from non-malignant origin. In this study, we first evaluated the detectability of CNAs in silico using our recently validated framework [19]. The simulations were based on the varying concentrations of (non-)melanoma-cell derived DNA of each individual sample, and corresponded well to the outcomes of in vitro experiments (Fig. 5). As a result, roughly half of the vfDNA+/UM+ samples were found to have a too low total DNA concentration and/or fraction of melanoma-cell derived DNA, and were mathematically underpowered to reliably detect chromosomal gains or losses with our proposed digital PCR experiments. While this means that no conclusions can be drawn on the presence of CNAs in these samples, this in silico analysis helped to avoid generating false-negative results and prevented an unnecessary waste of precious material.

Within the cohort of vfDNA+/UM+ samples, most BSE mutations and CNAs known to be present in the primary tumour were successfully identified in the vitreous fluids as well. However, a small number of cases was discordant (Fig. 2 and Supplementary Table 1).

Concerning the mutations, our repeated analysis of initially negative experiments proved that subsampling might be a main reason why lowly abundant mutations are not detected in each individual experiment: when few mutant alleles are present, they may be missed when only part of the specimen is analysed. Repeating the experiment may help in the identification of these rare mutant alleles (Supplementary Figs. 1 and 2). As another explanation, various mutation assays might have different sensitivity levels. Although we included negative control experiments (which turned out to be consistently negative, in line with a high specificity, Fig. 4 and Supplementary Fig. 1C), we did not compare all digital PCR mutation assays for their exact lower limits of detection. This would be a valuable and relevant addition to be tested in a clinically-oriented follow-up study.

In contrast, the discordant vitreous fluid samples that lacked detectable CNAs were predicted to have sufficient melanoma-cell derived DNA concentrations to detect chromosomal gains and losses, assuming that the alteration was clonally abundant in the entire fraction of melanoma-cell derived vfDNA. Intriguingly, all these cases demonstrated heterogeneity for this chromosomal loss or gain in the primary tumour (Supplementary Fig. 3). This admixed presence of clones with and without a certain alteration may explain why it could not be detected in the vitreous fluid: it may be not present at all or only present in an undetectable low proportion in the fraction of melanoma-cell derived vfDNA. These cases illustrate the analytical challenge of detecting all relevant alterations in heterogeneous samples.

From a clinical perspective, we found that the detectability of melanoma-cell derived vfDNA was associated with a higher amount of non-tumour cell derived vfDNA and a larger prominence of the primary tumour (Fig. 6A and C). This suggests that larger (and possibly more invasive) growth of uveal melanomas contributes to the presence of DNA in the vitreous fluid, originating from both melanoma and other cells. However, the identification of melanoma-cell derived vfDNA itself did not mark a genetically distinct or prognostically-relevant subgroup of patients (Fig. 6D and E). By combining the BSE mutation and CNA results, in 29/65 (45%) of the cases the molecular subtype of the primary tumour could be successfully inferred from the vfDNA measurements. Within this subgroup, the correlation between this molecular classification of tumours and patients’ risk of developing lethal metastases was clearly demonstrated (Fig. 6F).

Our findings illustrate that in a proportion of patients, relevant molecular information from their primary uveal melanoma may be derived from analysing the DNA present in the vitreous fluid. Following this approach, the risk of metastatic tumour dissemination may be estimated without a direct tumour biopsy, and an increasing number of patients could be offered a personalised prognosis and concordant clinical follow-up. In the future, knowledge about the genetics of a patient’s tumour might also be relevant for (neo-)adjuvant or targeted therapies [5, 14, 15].

Nevertheless, it is crucial to note that in our current study, vitreous fluid samples were investigated that had been collected ex vivo from enucleated eyes. This procedure is different to how it would be in a clinical setting, and uveal melanomas treated by enucleation are typically larger and more invasive than irradiated tumours. Therefore, a follow-up study should evaluate the applicability of our methodology in a prospective clinical context, for example by defining patient groups most likely to benefit from the analysis (e.g. those with relatively thick tumours), and by evaluating vfDNA collected in a similar way as performed in vivo from non-enucleated eyes.

One may also consider to explore possible additions and alternatives to our current methodologies. For example, epigenetic targets (such as DNA methylation) may be evaluated as markers for the various molecular subtypes of uveal melanomas. Candidate loci have already been identified [29, 30], and as DNA methylation can be measured using digital PCR as well [31, 32], the analysis may be easily incorporated in our current workflow. Additionally, the experimental performance might be optimised to lower its limits of detection, for example by denaturating the DNA samples prior to digital PCR analysis [33]. As further alternatives, vitreous fluid samples may be studied using complementary molecular techniques (such as ultradeep sequencing) or by analysing its RNA, as we recently showed that tumour-derived genetic information may also be inferred from a transcriptional level [34].

Lastly, we believe that our digital PCR assays and approaches have translational potential in the analysis of heterogeneous solid or liquid uveal melanoma biopsies of any other origin, and beyond the context of uveal melanoma. Some of the mutation assays described in this study have already been used in blood-based screening of uveal melanoma patients [35], and the value of similar applications is increasingly recognised [36]. Moreover, the innovative methods we successfully applied to sensitively analyse scarce specimens (e.g. the drop-off and SNP-based assays, as well as the in silico and multiplex setups) may be relatively easily modified to be used in other malignancies.

## Conclusions

Our findings demonstrate the possibility of capturing the molecular profile of a primary uveal melanoma by analysing the DNA present in the vitreous fluid of the eye. As this approach does not require a biopsy to be taken from the tumour itself, a molecular classification of the primary tumour and concordant personalised prognosis and follow-up might be offered to more patients. In the end, this may ameliorate the clinical outcome of patients with this aggressive malignancy.

## Electronic supplementary material

Below is the link to the electronic supplementary material.

Supplementary Material 1

Supplementary Material 2

## Data Availability

All processed results of the dataset analysed in this study are available in Supplementary Table 1. All custom scripts are available via https://github.com/rjnell/um-vitreous. Raw data are available from the corresponding author upon reasonable request.

## Abbreviations

BSE mutation: Mutation affecting BAP1, SF3B1 or EIF1AX
CNA: Copy number alteration
Gα_q_ (signalling) mutation: Mutation affecting *GNAQ*, *GNA11*, *CYSLTR2* or *PLCB4*
LUMC: Leiden University Medical Center
SNP: Single-nucleotide polymorphism
vfDNA: DNA isolated from vitreous fluid
vfDNA−/UM−: Sample not containing any measurable levels of DNA
vfDNA+/UM−: Sample containing measurable levels of DNA without uveal melanoma-derived DNA
vfDNA+/UM+: Sample containing measurable levels of DNA including uveal melanoma-derived DNA
