# Supplementary material for: Digital PCR-based genetic profiling from vitreous fluid as liquid biopsy for primary uveal melanoma: a proof-of-concept study
Source: J Exp Clin Cancer Res. 2025 Apr 17;44:124. doi: 10.1186/s13046-025-03374-y (PMC12004579; doi:10.1186/s13046-025-03374-y)
Supplement: Supplementary file 1 — Supplementary Material 1 [file 13046_2025_3374_MOESM1_ESM.docx]

# Supplementary Information

## Supplementary Table 1

Overview of clinical and molecular information of all cases analysed in this study.

## Supplementary Figure 1


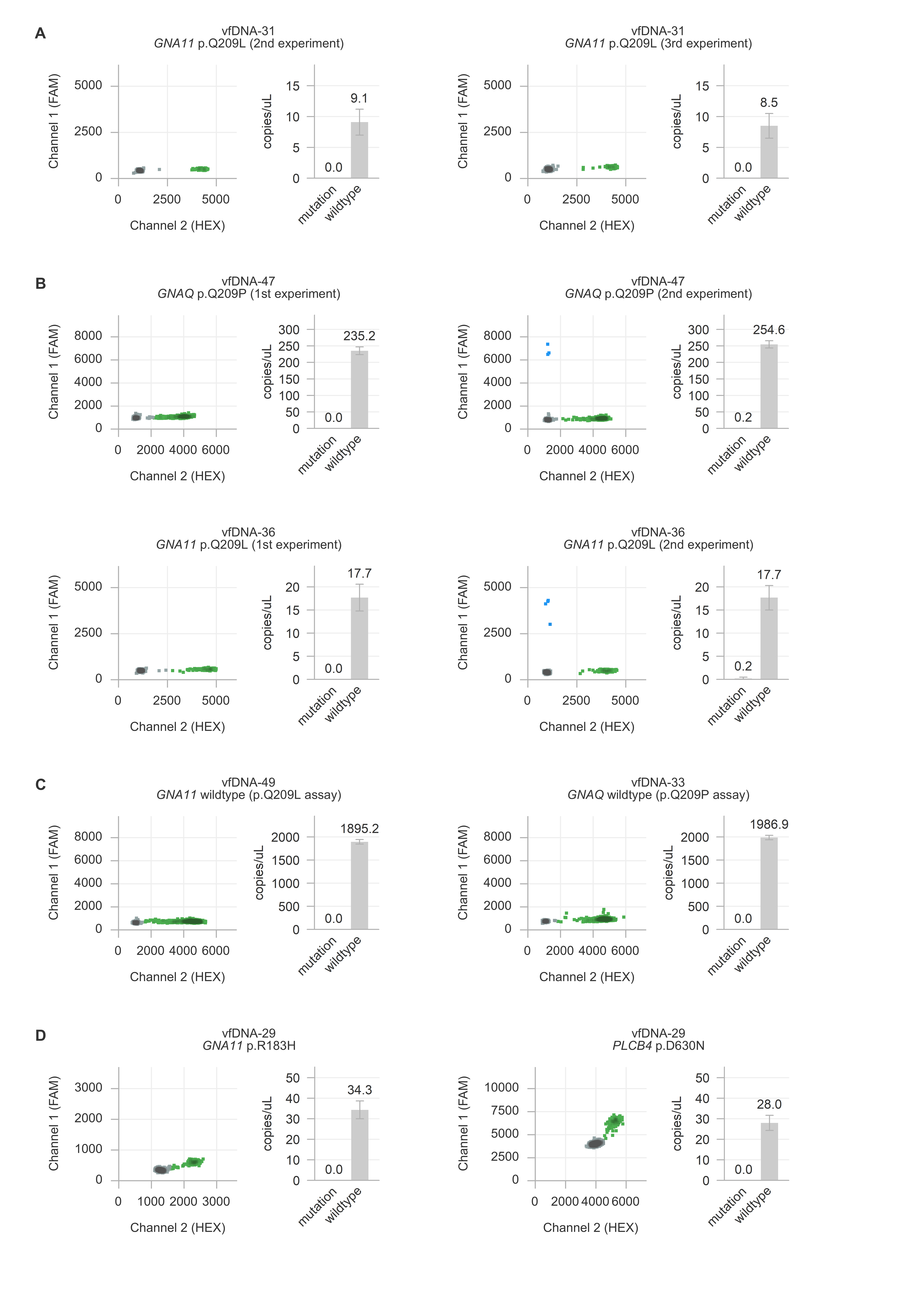


Analysis of Gα_q_ signalling mutations using targeted digital PCR. The colour of the cluster indicates the content of the digital PCR droplets (grey: no target DNA, blue: mutant DNA, green: wild-type DNA, orange: wild-type and mutant DNA).

(**A**) Repeated analysis of the *GNAQ* p.Q209L wild-type and mutant alleles in vfDNA-31 (the first experiment is presented in **Figure 2A**), not revealing any mutation-positive droplets.

(**B**) Repeated analysis of the *GNAQ* p.Q209P wild-type and mutant alleles in vfDNA-47, and *GNA11* p.Q209L wild-type and mutant alleles in vfDNA-36. For both cases, a low number of mutation-positive droplets is detected in the second, but not the first experiment.

(**C**) Negative control experiments for the *GNA11* p.Q209L and *GNAQ* p.Q209P assays not revealing any mutation-positive droplets in wild-type vfDNA samples.

(**D**) vfDNA-29 was measured for two Gα_q_ signalling mutations due the presence of both *GNA11* p.R183H and *PLCB4* p.D630N mutations in the matched primary tumour. The vfDNA only contained wild-type *GNA11* and *PLCB4* in comparable concentrations.

## Supplementary Figure 2


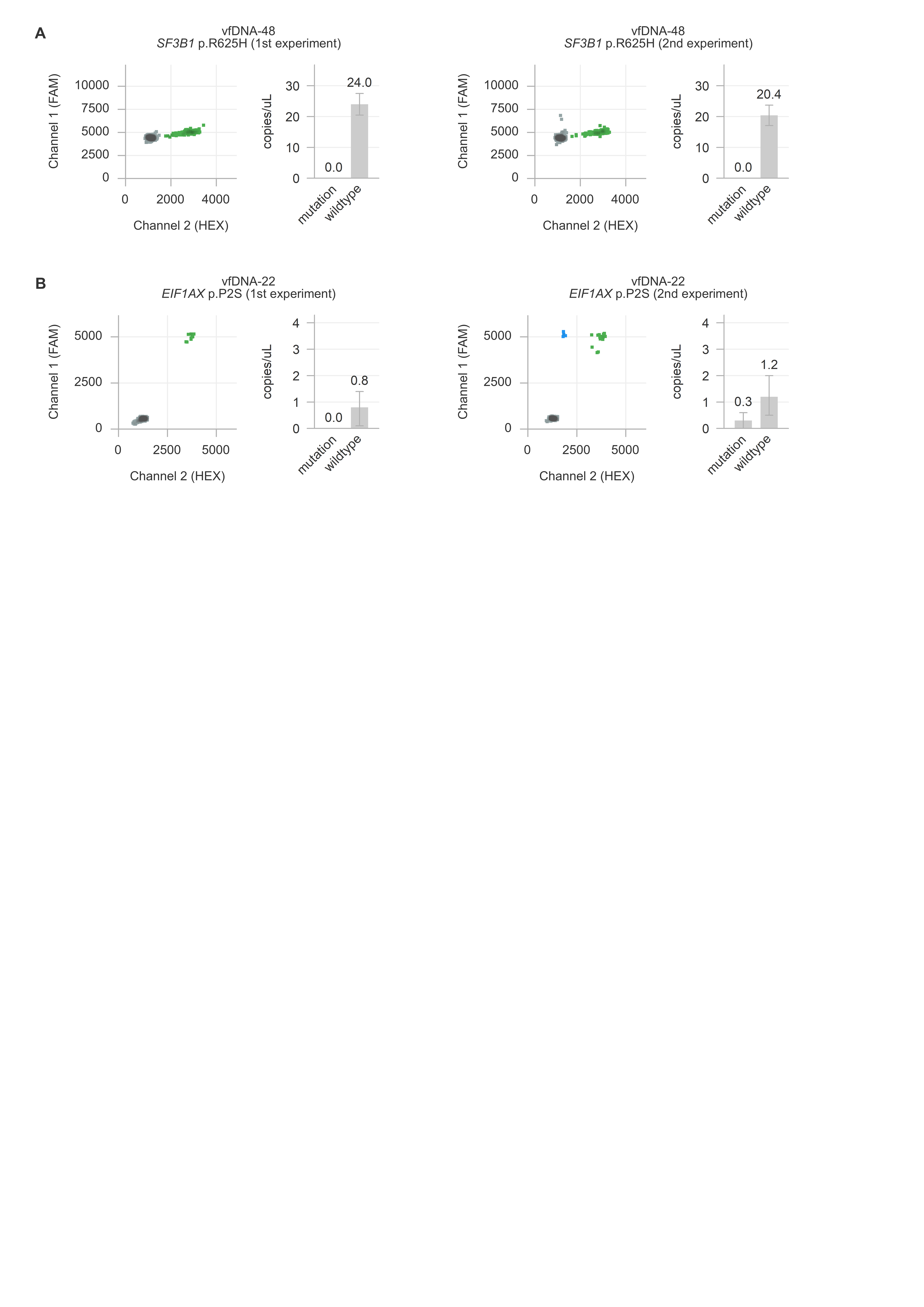


Repeated analysis of two BSE mutations using targeted digital PCR. The colour of the cluster indicates the content of the digital PCR droplets (grey: no target DNA, blue: mutant DNA, green: wild-type DNA, orange: wild-type and mutant DNA).

(**A**) Repeated analysis of the *SF3B1* p.R625H wild-type and mutant alleles in vfDNA-48, not revealing any mutation-positive droplets.

(**B**) Repeated analysis of the *EIF1AX* p.P2S wild-type and mutant alleles in vfDNA-22. A low number of mutation-positive droplets is detected in the second, but not the first experiment.

## Supplementary Figure 3


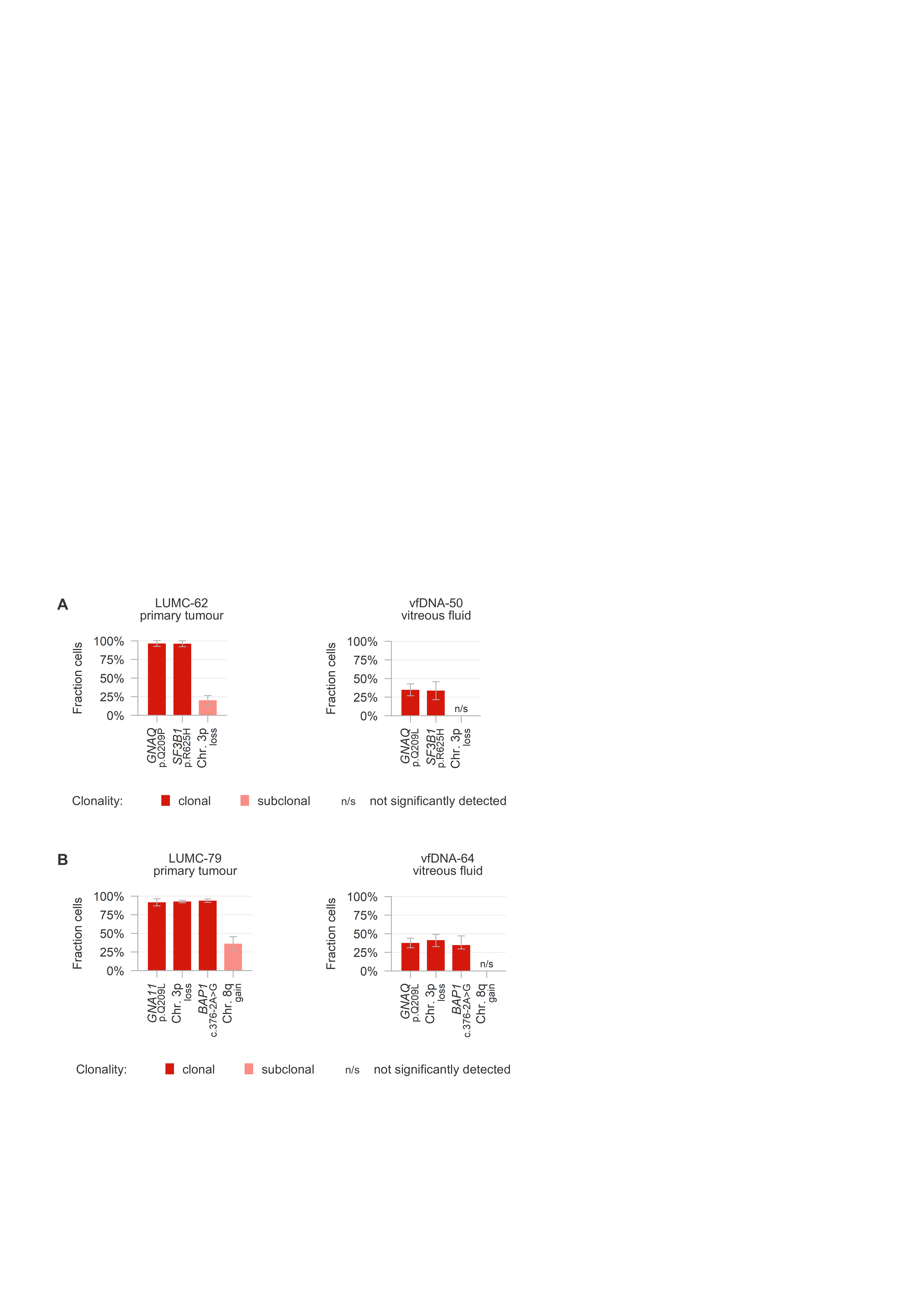


(**A**) and (**B**) Clonality analyses of matched primary tumour and vitreous fluid in two patients, based on the data from reference (20) and the current study. In both patients, a variety of alterations are clonally present in both the primary tumour and vitreous fluid. However, the loss of chromosome 3p in patient (**A**) and the gain of chromosome 8q in patient (**B**) are subclonally present in the primary tumour and not significantly detected in the vitreous fluid.
